# 3D Reconstruction and Prediction of Sialolith Surgery

**DOI:** 10.1155/2018/3951956

**Published:** 2018-11-13

**Authors:** Jamyson Oliveira Santos, Brunna da Silva Firmino, Matheus Santos Carvalho, Jean de Pinho Mendes, Lucas Novaes Teixeira, Sérgio Lúcio Pereira de Castro Lopes, André Luiz Ferreira Costa, Antonione Santos Bezerra Pinto

**Affiliations:** ^1^State University of Piauí, Parnaíba, Brazil; ^2^Surgery and Clinic Integrated Department, State University of Piauí, Parnaiba, Brazil; ^3^Department of Oral Pathology, São Leopoldo Mandic Institute and Research Center, Campinas, São Paulo 13045-755, Brazil; ^4^Department of Diagnosis and Surgery, Institute of Science and Technology, State University of São Paulo (UNESP), São José dos Campos, Brazil; ^5^Department of Orthodontics and Radiology, University of São Paulo City, São Paulo, Brazil; ^6^Department of Morphology, Faculty of Medicine, Federal University of Ceará, Fortaleza, Brazil

## Abstract

Imaging examinations play an important role in the diagnosis of sialolithiasis, whose symptoms are initially confounded with other diseases. The objective of the present case report is to highlight imaging and processing techniques as well as image analysis for the preoperative assessment and planning of surgical interventions and adequate treatment of massive sialoliths. A 35-year-old male patient presented complaining of pain in the submandibular region and purulent secretions from a lingual caruncle with slightly increased volume in the region. Imaging examinations were ordered as follows: cone beam computed tomography, ultrasonography, and three-dimensional reconstruction, including clinical evaluation. A final diagnosis of sialolithiasis was established. Surgery was indicated and carried out by using a lateral transcervical approach for complete resection of the gland, which was based on the calculation of the total volume of the sialolith, thus increasing the surgery's success.

## 1. Introduction

Sialoliths are calcified masses which occasionally affect the salivary glands [1, 2]. In general, they are asymptomatic, presenting a slight increase in glandular volume during salivary stimulation, which causes a mild sensation of discomfort [3, 4].

The aetiology of salivary calculi and the exact mechanism of their formation are still unknown. Nevertheless, aetiological factors may be related to the salivary content [2, 5], including other factors such as trauma to salivary duct or gland and precipitation of salts linked to certain organic substances. There is also a theory in the literature stating that a retrograde infection might explain the sialolithiasis as food residues, substances, or bacteria which can migrate into the salivary ducts, becoming then a niche for more calcification [3–6].

Several techniques are used for diagnosis of sialolithiasis, ranging from simple techniques such as palpation and inspection [2], including analysis of secreted saliva [2, 7], to complementary examinations such as radiography, sialography, computed tomography, and ultrasonography [1–7]. However, depending on the location of sialolith (e.g., mainly in the duct of the submandibular gland), occlusal radiograph can be used for its visualisation [3, 8] if it is located in the glandular parenchyma; otherwise, other imaging examinations allow a better visualisation. In this latter case, complete resection of the gland is necessary because there is a high rate of relapse and the calcified mass cannot pass through the duct, which requires a lateral transcervical approach for removal of the submandibular gland [2–4, 8, 9].

Three-dimensional reconstruction was performed because it is a useful and precise method for assessing the volume and morphological changes in the target structures [10, 11]. The aim of the present report is to describe a case of sialolithiasis and the 3D visualisation of the salivary calculus by using 3D reconstruction software, thus enabling a better understanding of the morphology of the lesion and its proximity to adjacent tissues, including ideal measurements for optimising the surgical access.

## 2. Case Report

**Figure 1:**
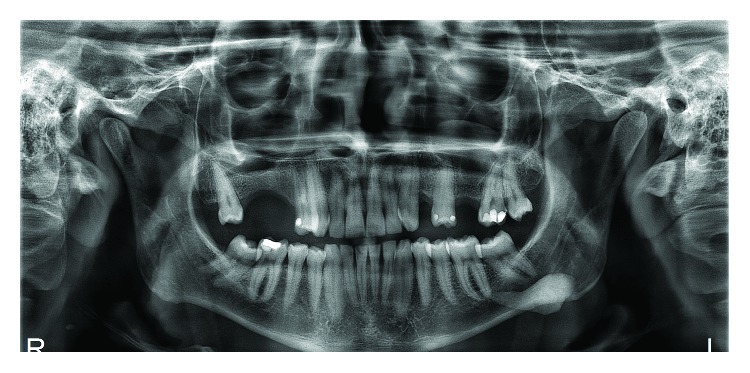
Panoramic radiography revealing radiopaque mass in the region of the left mandibular body.

**Figure 2:**
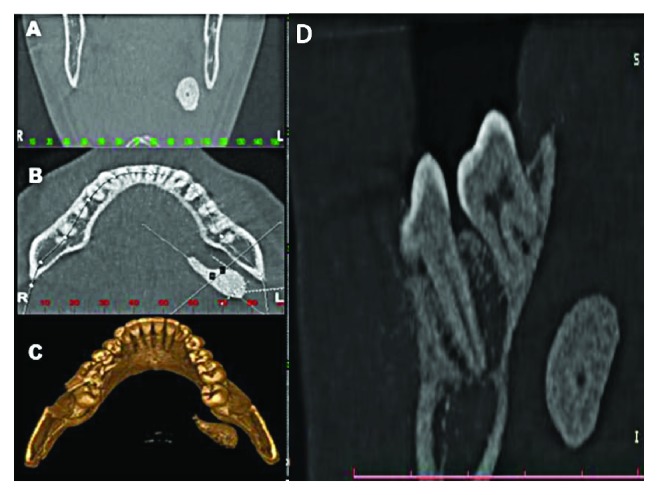
Cone beam computed tomography showing the dimensions of sialoliths. (a) Coronal view. (b) Axial view. (c) 3D reconstruction. (d) Sagittal view.

A 35-year-old white male patient presented complaining of pain in the submandibular region and purulent secretion from a lingual caruncle with slightly increased volume in the region. Panoramic radiography showed a dense radiopaque mass in the region corresponding to the left submandibular gland (Figure 1). However, plain radiographs are not appropriate for the diagnosis of calcified inflammatory diseases [12]. As a result of the magnification, geometric distortion, and superimposition of structures on the panoramic radiography, cone beam computed tomography (CBCT) was used to evaluate the lesion. The CBCT showed a hyperdense image of 24.35 mm × 9.35 mm (Figure 2).

Ultrasound is usually used as the first exam to evaluate the salivary glands because it is noninvasive and with low cost [13].

**Figure 3:**
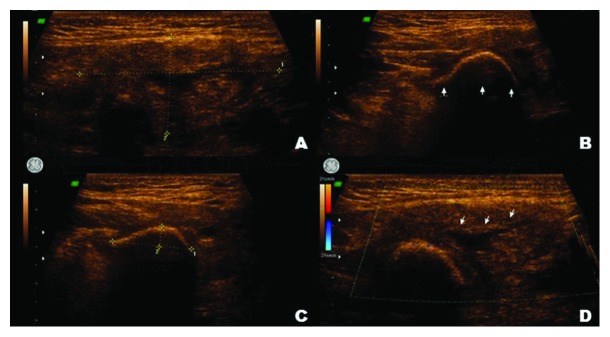
Ultrasonograph of the left submandibular gland showing (a) total dimensions of the gland, (b, c) sialolith and its size, and (d) dilatation of the duct.

Ultrasonography showed an increased volume of submandibular gland with diffuse echogenicity changes associated with salivary duct dilatation, viewed as a partially circumscribed hyperechogenic image measuring 1.9 cm × 1.1 cm × 0.5 cm located in the adjacent sublingual region (Figure 3).

**Figure 4:**
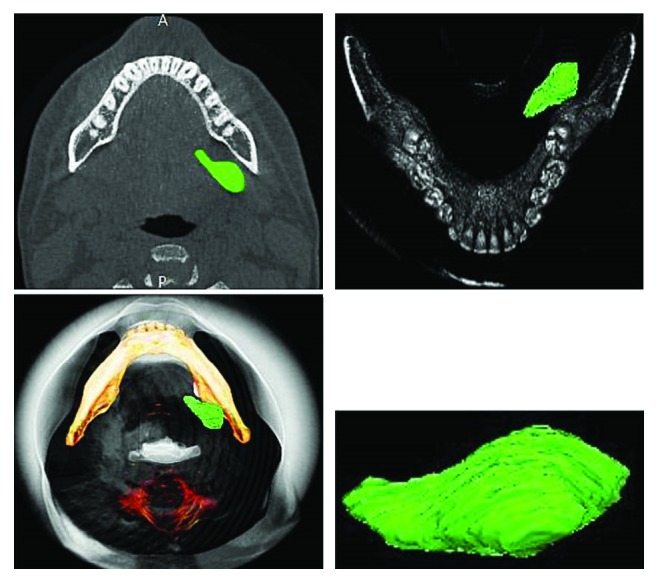
Three-dimensional segmentation for the calculation of the sialolith's volume by using the InVesalius software.

Prior to the surgical procedure, the InVesalius software (https://www.cti.gov.br/pt-br/invesalius#download) was used to assess the ratio between sialolith's volume and submandibular gland involved for analysis of gland resection. After 3D reconstruction, it was possible to reduce bone transparency and observe the morphology of sialolith, which was isolated from other structures in order to allow the evaluation of its area, volume, and position. CBCT image shows the area corresponding to the sialolith, which was manually segmented (Figure 4). The addition of volume (123 mm^3^) provided us a new perspective regarding not only the extension and size of the lesion (in mm^3^) but also the analysis of morphological aspects of the salivary calculus, thus confirming the need of surgical treatment.

**Figure 5:**
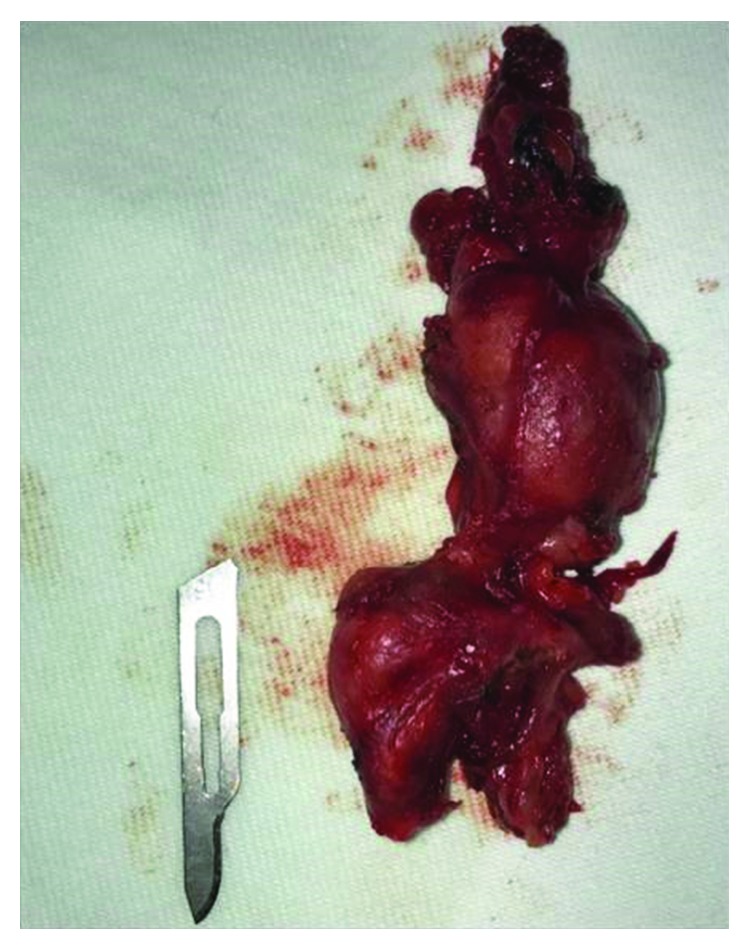
Fragment of tissue collected, with the lobular portion next the scalpel corresponding to the sialolith's area.

After indication for surgery, the patient was placed on supine position with the left side of the neck exposed, showing the upper cervical skin 4 cm below the mandibular angle, where incision and opening of the flap were performed with an electrocautery device superficially placed on the platysma muscle. Next, the mandibular and cervical rami of the facial nerve were located, dissected, and protected. The posterior facial vein was clamped and sectioned, with the stump being isolated in order to facilitate the separation of the mandibular ramus cranially along with the upper skin flap. Next, the soft parts of the submandibular gland were dissected, exposing the anterior belly of the digastric muscle before sectioning of the small vessels to allow mobilisation of the gland and its complete resection. A fragment of soft tissue measuring 70 mm × 28 mm × 17 mm having irregular surface, elongated shape, blackened colour, and fibrous consistency was collected (Figure 5).

**Figure 6:**
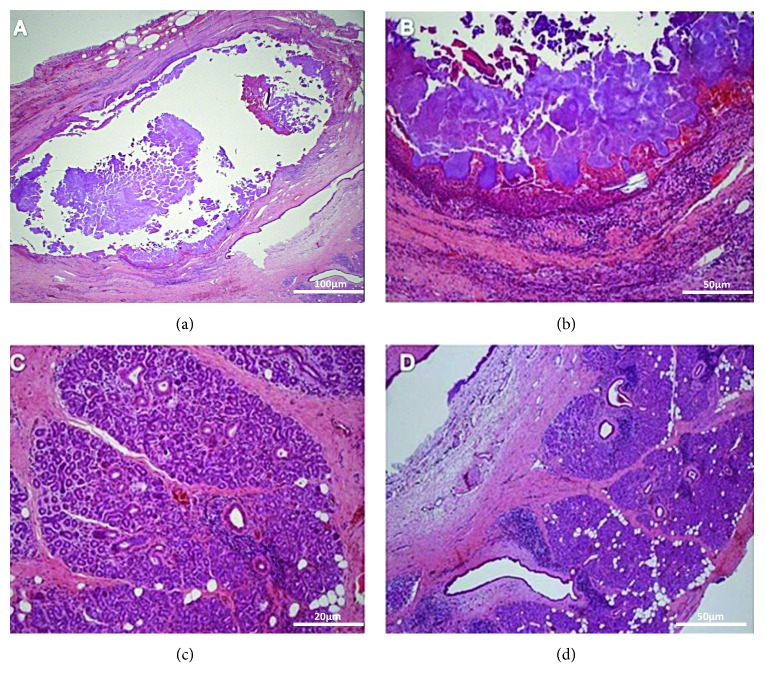
H&E histological sections of fragments of salivary gland lobules centrally showing excretory duct containing granular basophilic material and mineralised areas with a concentric deposition pattern compatible with sialolith, sialolithiasis, and intercalated duct lesion.

Microscopic examination revealed fragments of salivary gland lobules showing excretory duct containing granular basophilic material and mineralised areas with a concentric deposition pattern compatible with a sialolith. In other areas, one can observe stroma composed of dense connective tissue exhibiting intense lymphoplasmacytic inflammatory infiltrate and congested blood vessels. From within, it was possible to identify areas showing salivary gland lobules, with the majority presenting acini replaced by the proliferation of duct-shaped structures exhibiting a double-layer of cells among the scant amount of connective tissue. In some areas, the proliferation of duct-shaped structures was found with intense lymphoplasmacytic inflammatory infiltrate. In the focal area, one could also observe the presence of exogenous material of yellowish colour and refractive aspect, exhibiting strange body reaction characterised by the presence of multinucleated giant cells (Figure 6).

In the postoperative period, the patient reported paresthesia in the region of the lingual nerve. Therefore, a protocol with low laser therapy with an energy per point from 1.0 to 4.0 joules was applied. After this procedure, a considerable improvement in paresthesia was observed and the evolution of the cicatrization process was also accelerated allowing the patient to recover the regular movements of the neck, previously limited by edema. The residual scar was satisfactory and did not significantly compromise the aesthetics of the patient.

## 3. Discussion

The aetiology of sialolithiasis is still unknown, but some authors believe that persistent changes in saliva flow along with an increased amount of mucus facilitates the precipitation of amorphous phosphate tricalcium [2, 3, 5, 8], which becomes crystallised and transforms into hydroxyapatite. From this matrix, there is an apposition of several substances which also become calcified and form the sialolith [2, 3, 5, 6, 8].

Statistical data cited elsewhere reveal that sialolithiasis corresponds to more than 50 percent of the major salivary gland diseases. The great majority (80–90 percent) of salivary calculus occur in the submandibular gland, whereas 5–20 percent in the parotid gland [1–7] and in the sublingual gland. As for the minor salivary glands, the occurrence of calculus is less common. Sialolithiasis may occur at any age, in general, emerging between 30 and 60 years old. Sialolithiasis in children is rare, tending to occur more frequently in men than in women [2–6, 8].

According to the literature, the painful symptoms affect the patients mainly during salivary stimulation [1–3, 6–8] and, in general, this event makes them seek professional help. For Ligtenberg and Veerman, imaging findings of calcifications in large salivary glands and/or salivary ducts are usually related to salivary calculus or sialoliths [14]. Therefore, based on such findings, it is indispensable to perform clinical examination and anamnesis [1, 3, 5, 7, 8, 11, 14].

There are several diagnostic imaging tools available which are aimed at improving investigation, namely, panoramic radiography, occlusal radiography, sialography, ultrasound, and computed tomography, especially cone beam computed tomography and magnetic resonance [1, 4, 7, 11, 14]. Although only 20 percent of the normal panoramic radiographs and occlusal radiographs are capable of evidencing sialoliths [14], they are valid examinations as they allow excluding mandibular bone pathologies resembling this condition. Sialography is a technique in which a contrast solution is injected into the glandular duct. Cone beam computed tomography allows a better evaluation of the glandular parenchyma and adjacent structures involved, thus being possible to obtain precise information for choosing the best treatment. Ultrasonographic examination, which is more used for cystic lesions (e.g., ranulas), can also be a valued tool for obtaining information on the size and shape of calcifications and their exact localisation [1, 7, 12–14].

A surgical focus facilitating the removal of the lesion safely and precisely, thus decreasing the patient's morbidity and preventing injuries to adjacent tissues, should be the main objective of all operative procedures [15, 16]. With this aim, previous planning and access direction become one of the key principles for removal of the lesion, that is, the more precise it is, the better the procedure.

Image volumetry is a fundamental stage in many conditions in medical image-based analysis and treatment planning tasks [17]. Current works have provided a growing concern in using this 3D reconstruction adding detailed spatial/structural information for the presurgery of ghost cell odontogenic carcinoma, renal tumors, and other lesions [17–19].

Tomographic images can be processed into 3D images, providing a better spatial idea of the anatomy and leading to an effect similar to anatomical structures and the own operative condition to be considered [17]. In the present case report, we have shown 3D images provided by the InVesalius software, which demonstrated the importance of 3D reconstruction in the surgery planning by giving the surgery team more information on the shape and location of the lesion, since both aspects may not be clearly observed on 2D images or individualised on CT images. Consequently, precise spatial and structural information (e.g., volume index) can be added to the surgery planning for long-term assessment of the surgical outcome. Open-source software, as that used in the present case, offers a valued tool for virtual analysis at low cost and unrestricted access, thus playing an important role in the diagnosis and serving as an intraoperative guide prior to surgery [17].

Resection of the submandibular gland was the treatment chosen for solving the present clinical case, since intraoral access for removal of the calculus was unfeasible due to its dimensions [2, 4, 8]. A lateral transcervical approach was used, and according to De Carvalho et al., it is considered the standard surgical access for submandibular gland [9].

For sialoliths closer to duct ostia, the treatment can be conservative, using saliva stimulants and acidic foods to stimulate the production of saliva until the elimination of the sialolith. It is also possible to choose the catheterization or dilatation of the duct for calculus extraction. For sialoliths located in the anterior half of the duct, surgical intervention is required for the removal of sialoliths, mainly by intraoral surgery [4, 9]. In cases when the calculus is located in the posterior portion of the duct or inside the gland, the approach is always surgical and may be associated with the total removal of the gland, such as in our case, where the treatment of choice was to remove the sialoliths, followed by excision of the gland (indicated on volumetric analysis) [9]. Caution is necessary for surgical treatment, as it can lead to complications such as fibrosis in the ductus, salivary fistula, paralysis, and paresthesia. There are two more recent methods that can also be used as a form of treatment: endoscopy, which is used for massive salivary calculus, such as the region near the lingual nerve, and lithotripsy, which consists of a focused, extracorporeal shock wave through the skin to the calculus and which fragments the calculus and decreases its size [8, 9].

In the immediate postoperative period, the patient presented mild paresthesia in the region of the lingual nerve that was successfully treated with low laser therapy. The follow-up examination 15 days later was uneventful, and the patient recovered without any complication. Therefore, 3D reconstruction allowed the sialoliths' extent and analysis of the sialolith volume increasing the accuracy of the surgical management.

## 4. Conclusion

The present study shows that 3D reconstruction is convenient as an assisting tool in the preoperative planning, thus optimising the surgical access and facilitating the recognition of the lesion's shape for volumetric analysis. Therefore, this can provide the surgery team more precise information in order to help in the surgery planning, thus increasing the likelihood of treatment success.
